# Brief Visual Deprivation Selectively Remodels Intracortical Inputs onto Layer 6 Corticothalamic Neurons in Primary Auditory Cortex

**DOI:** 10.1523/ENEURO.0205-26.2026

**Published:** 2026-10-09

**Authors:** Anthony T. Collins, Joseph P. Y. Kao, Hey-Kyoung Lee, Patrick O. Kanold

**Affiliations:** ^1^Solomon H. Snyder Department of Neuroscience, Johns Hopkins University School of Medicine, Baltimore, Maryland 21205; ^2^Zanvyl-Krieger Mind/Brain Institute, Johns Hopkins University, Baltimore, Maryland 21218; ^3^Center for Biomedical Engineering and Technology, and Department of Physiology, University of Maryland School of Medicine, Baltimore, Maryland 21201; ^4^Kavli Neuroscience Discovery Institute, Johns Hopkins University School of Medicine, Baltimore, Maryland 21205; ^5^Department of Biomedical Engineering, Whiting School of Engineering and Johns Hopkins University School of Medicine, Baltimore, Maryland 21205

**Keywords:** auditory, corticothalamic, mouse, plasticity, visual deprivation

## Abstract

Loss of one sensory modality can enhance processing in spared systems. Brief visual deprivation by dark exposure (DE) enhances auditory responses in the primary auditory cortex (A1) of adult mice, increasing firing rates, frequency selectivity, and behavioral performance. This enhancement is accompanied by reduced inhibition of the auditory thalamus (MGBv) and strengthened thalamocortical and intracortical feedforward inputs. While the mechanisms coordinating cross-modal cortical and thalamic plasticity are unknown, Ntsr1+ layer 6 corticothalamic neurons (L6-CTNs) are a promising candidate given their regulation of both cortical and thalamic gain. Here, we examined how 1 week of DE in both male and female adult mice alters intracortical synaptic inputs onto A1 L6-CTNs using laser-scanning photostimulation and whole-cell recordings. We found DE selectively reduced excitatory intracortical connectivity onto L6-CTNs across multiple layers and weakened synaptic strength from infragranular inputs. In contrast, inhibitory inputs were largely preserved. This shift resulted in a layer-specific reduction in excitation–inhibition balance, particularly for inputs from layer 4. Although the spatial extent of inputs was unchanged, DE decreased the within-animal similarity of excitatory input patterns across neurons, indicating a reorganization of circuit structure. Together, these findings identify selective weakening of intracortical excitation onto L6-CTNs as a key feature of cross-modal plasticity. By reducing recruitment of the L6 gain-control circuit, this reconfiguration may bias cortical processing toward enhanced sensory discrimination while preserving sensitivity.

## Significance Statement

Sensory deprivation induces thalamic and cortical plasticity that can enhance processing in spared sensory systems. Layer 6 corticothalamic neurons (L6-CTNs) regulate cortical and thalamic activity, but how deprivation alters their circuitry is unknown. Using laser-scanning photostimulation in adult mouse auditory cortex, we found that brief visual deprivation selectively reduced the area and strength of intracortical excitatory inputs onto L6-CTNs while largely preserving inhibition. This reconfiguration shifted excitation–inhibition balance toward inhibition and reduced within-animal similarity of excitatory input patterns. These findings identify weakening and reduced stereotypy of intracortical excitation onto L6-CTNs as features of cross-modal plasticity, suggesting that remodeling of the L6 feedback circuit may coordinate plasticity across cortical and thalamic components of a spared sensory system.

## Introduction

Sensory loss or deprivation can lead to enhanced function of the spared senses. For example, congenitally deaf individuals exhibit enhanced visual attention in their peripheral visual field (Bavelier et al., 2000), whereas blindness is also associated with improved tactile spatial acuity, demonstrating that compensatory enhancement can extend to somatosensory processing (Goldreich and Kanics, 2003). Furthermore, heightened auditory abilities are often reported by blind individuals, including enhanced auditory sensitivity and discrimination (Lessard et al., 1998; Röder et al., 1999; Gougoux et al., 2004). Such behavioral enhancements are also seen in animal models. Congenitally deaf cats have shown enhanced peripheral visual localization and visual motion detection (Lomber et al., 2010), and temporary visual deprivation (dark exposure, DE) in mice is associated with enhanced sound frequency sensitivity, sensory gain, and increased performance on auditory detection tasks (Petrus et al., 2014; Jendrichovsky et al., 2024). Underlying these adaptations are functional circuit changes in the spared sensory cortical regions, including both thalamocortical and intracortical synaptic plasticity.

While auditory thalamocortical plasticity is widely considered to be restricted to a brief developmental critical period in mice (Crair and Malenka, 1995; Hensch, 2004; Takesian et al., 2018), 1 week of DE in adults leads to stronger synaptic connections between the auditory thalamus (ventral portion of the medial geniculate body, MGBv) and L4 of the primary auditory cortex (A1; Petrus et al., 2014). This enhanced synaptic thalamocortical excitation is accompanied by a reduction of synaptic inhibition from the thalamic reticular nucleus (TRN) onto the MGBv (Whitt et al., 2022). Together, these DE-induced forms of thalamic plasticity bias auditory processing toward enhanced feedforward drive into A1, a circuit configuration typically associated with increased sensory gain and improved sound detection.

DE also impacts intracortical circuits within and between A1 layers 4 and 2/3. DE results in both remodeling and refinement of intracortical inputs onto L4 pyramidal cells from L2/3, as well as refinement of intracortical inputs onto L2/3 from L2/3 and L4, reflected by a reduction of excitatory and inhibitory input strength (Meng et al., 2015, 2017a). These coordinated changes and behavioral enhancements suggest that DE does not simply amplify auditory input but reorganizes cortical circuit functionality in a manner compatible with improved perceptual discrimination.

Given that auditory perceptual salience is the direct result of auditory sensory processing ability (shaped by the source, timing, and balance of excitatory and inhibitory inputs; Froemke et al., 2013; Pinto et al., 2013; Sohoglu and Chait, 2016), cortical circuits involved in sensory gain control likely play a crucial role in enabling this DE-enhanced auditory performance.

One such gain-control circuit originates in layer 6 (L6) and is mediated by corticothalamic projection neurons (L6-CTNs). Within a given primary sensory cortex (e.g., V1, S1, or A1), L6-CTNs are excitatory neurons that receive sensory information directly from the corresponding primary sensory thalamus (Bortone et al., 2014; Guo et al., 2017; Frandolig et al., 2019), while also integrating intracortical inputs spanning from deep to superficial layers (Vélez-Fort et al., 2014; DeNardo et al., 2015). In turn, L6-CTNs provide cortical feedback to their modality's primary sensory thalamus (MGBv, in the case of A1) and the inhibitory TRN, thereby regulating the excitatory/inhibitory balance of feedforward thalamocortical signaling. Notably, L6-CTNs promote TRN-mediated thalamic inhibition during low-frequency firing but facilitate thalamic output during high-frequency firing, positioning them as state-dependent regulators of thalamic sensory gating (Guo et al., 2017).

L6-CTNs also directly modulate cortical gain through monosynaptic excitatory projections onto L4 and L5 and disynaptic excitation of L2/3 (Kim et al., 2014). Furthermore, their collateral excitatory projections onto L6 parvalbumin-positive (PV) interneurons produce cortical-layer-wide disynaptic inhibition (Bortone et al., 2014; Guo et al., 2017; Frandolig et al., 2019). Thus, experience-dependent changes in the intracortical input connectivity of L6-CTNs are ideally positioned to coordinate both thalamic and cortical plasticity in A1. Here, we report that dark exposure selectively reduces intracortical excitatory connectivity onto L6-CTNs across multiple cortical layers, reflected by decreased connection probability, reduced total excitatory input, and diminished synaptic strength, while inhibitory inputs are comparatively preserved. This reorganization results in a shift in the intracortical excitatory–inhibitory balance onto L6-CTNs, identifying a circuit-level mechanism through which sensory deprivation may recalibrate cortical gain control in the adult auditory cortex.

## Materials and Methods

### Animals

All animal procedures were approved by the Institutional Animal Care and Use Committee at Johns Hopkins University.

Ntsr1-Cre mice (GENSAT line GN220; Mutant Mouse Regional Resource Center stock #017266-UCD; RRID: IMSR_MMRRC:017266; Gong et al., 2007) were crossed with Ai14 reporter mice [B6.Cg-Gt(ROSA)26Sortm14(CAG-tdTomato)Hze/J; stock #007914, The Jackson Laboratory; RRID:IMSR_JAX:007914; Madisen et al., 2010] to visualize robust tdTomato labeling of layer 6 corticothalamic neurons (L6-CTNs; Ntsr1-tdT), as established by previous works (Bortone et al., 2014; Kim et al., 2014; Guo et al., 2017; Sundberg et al., 2018). All animals were housed in a normal 12 h light/dark cycle with food and water *ad libitum*. Both male and female mice, postnatal days (P) 45–65 at the date of electrophysiological recordings, were used. One week before electrophysiological recordings, animals of the same sex were randomly split into two groups: one group remained in the normal light/dark cycle (Normal reared; NR), and another group underwent dark exposure (DE; see below for details).

### Dark exposure

Between P38 and P58, mice were randomly assigned to two group-housing conditions. Mice that were experimentally visually deprived were placed into a completely light-tight dark room for 1 week (7 ± 1 d) in a process dubbed DE. Mice in the control group remained housed under normal rearing (NR) conditions of a 12 h light/dark cycle. All mice were housed alongside other litter members of the same sex. DE as a means of visual deprivation was used as a reversible, bilateral method of eliminating visually driven input throughout the 24 h cycle. Although constant darkness removes photic entrainment, healthy mice generally retain robust circadian behavioral rhythms under these conditions (Jendrichovsky et al., 2024), with an endogenous free-running period of ∼23.7 h (Richardson et al., 1985, 2023; Rijo-Ferreira et al., 2018). This period predicts a gradual phase advance of ∼18 min per day, rather than a loss of circadian rhythmicity. This procedure is consistent with our previous studies (Meng et al., 2015, 2017a).

### Slice preparation

Each mouse was deeply anesthetized using isoflurane vapors until the absence of corneal reflex and toe pinch response and then transcardially perfused with ice-cold *N*-methyl-d-glucamine (NMDG) cutting solution as follows (in mM): 92 NMDG, 2.5 KCl, 1.25 NaH_2_PO_4_, 30 NaHCO_3_, 20 HEPES, 25 glucose, 2 thiourea, 5 Na-ascorbate, 3 Na-pyruvate, 12 *N*-acetyl cysteine, 0.5 CaCl_2_, and 10 MgSO_4_, with pH adjusted to 7.4. They were immediately decapitated. The brain was removed and immersed in ice-cold NMDG cutting solution. Slices (500 µm thick) were cut ∼15° from the horizontal plane to contain both A1 and the MGB, as well as maintain the tonotopic organization in the slice (Cruikshank et al., 2002; Zhao et al., 2009; Meng et al., 2015, 2017a). Slices from the left hemisphere were collected and placed in a small chamber filled with NMDG solution maintained at 30°C and bubbled with carbogen (95% O_2_/5% CO_2_) and incubated for ∼8 min before being transferred to room temperature ACSF (in mM): 124 NaCl, 5 KCl, 1.25 NaH_2_PO_4_·H_2_O, 26 NaHCO_3_, 10 dextrose, 2.5 CaCl_2_, and 1.5 MgCl_2_, bubbled with 95% O_2_/5% CO_2_ for 1 h.

### Electrophysiological recordings

Whole-cell recordings were performed as previously (Meng et al., 2015, 2017a) with a patch-clamp amplifier (MultiClamp 700B, Molecular Devices) using pipettes with a resistance of 4–9 MΩ. L6-CTNs were visualized with a digital camera (QImaging Retiga 3000 Snapshot CCD Camera) using both infrared differential interference contrast (IR-DIC) microscopy and epifluorescence. Pyramidal cells targeted for recording were in an area of A1 overlying the rostral flexure of the hippocampus. Data acquisition was performed by National Instruments AD boards and custom software: Ephus (Suter et al., 2010), written in MATLAB (MathWorks) and adapted to our setup. Voltages were corrected for an estimated junction potential of 10 mV. Recordings were performed at room temperature, and caged glutamate [0.8 mM *N*-(6-nitro-7-coumarylmethyl)-ʟ-glutamate; Muralidharan et al., 2016 ] was added in high-divalent ACSF to reduce the probability of polysynaptic inputs (in mM): 124 NaCl, 5 KCl, 1.25 NaH_2_PO_4_·H_2_O, 26 NaHCO_3_, 10 dextrose, 4 CaCl_2_, and 4 MgCl_2_, bubbled continuously with 95% O_2_/5% CO_2_. Electrodes were filled with an internal solution containing the following (in mM): 115 cesium methanesulfonate (CsCH_3_SO_3_), 5 NaF, 10 EGTA, 10 HEPES, 15 CsCl, 3.5 MgATP, and 3 QX-314, pH 7.25 (300 mOsm). Only cells exhibiting series resistance ≤25 MΩ were used for analysis.

### Laser-scanning photostimulation

Laser-scanning photostimulation (LSPS) was performed as described previously (Meng et al., 2015, 2017a; Xu et al., 2025). Laser stimulation (1 ms) was delivered through a 10× water immersion objective (Olympus). Laser power on the specimen was <25 mW and was held constant between recordings. The power was chosen based on prior experiments resulting in reliable activation across cortical layers (Meng et al., 2015, 2017a; Xu et al., 2025). For each map, an array of up to 30 × 30 sites with 40 µm spacing was stimulated once at 1 Hz in a pseudorandom order. This stimulation paradigm evokes an action potential at the stimulation sites with similar spatial resolution (∼100 µm) over cells in all cortical layers (Meng et al., 2015, 2017a; Xu et al., 2025). Thus, we spatially oversample at ∼3× in each dimension. Putative monosynaptic excitatory postsynaptic currents (EPSCs) were classified by the poststimulation latency of the evoked current. Evoked currents with latencies of <8 ms are likely to be the result of direct activation of glutamate receptors on the patched cell. Evoked currents with latencies between 8 and 50 ms were classified as monosynaptic evoked EPSCs. The first peak amplitude and the charge (the area of the EPSC in the counting window) were quantified for each synaptic response. Cells that did not show any large (>100 pA) direct responses were excluded from the analysis, as these could be astrocytes. Excitatory and inhibitory synaptic currents were recorded at −70 and 0 mV, the reversal potentials for GABA and glutamate, respectively. Prior cell-attached recordings under identical photostimulation conditions have demonstrated that neurons across cortical layers, including L6a and L6b populations, reliably fire action potentials in response to photo-released glutamate and that the spatial resolution and opto-excitability of LSPS remain stable across development and experimental conditions (Viswanathan et al., 2012; Meng et al., 2014, 2015, 2017a,b, 2021; Xue et al., 2023).

### Data analysis

Data analysis was performed using a combination of MATLAB, GraphPad Prism, and R (https://www.r-project.org) programs. LSPS maps were analyzed from individual recorded cells and grouped by experimental condition, input layer, cell identity, and animal identity.

Layer boundaries were identified by features in the bright-field image, e.g., cell density, cell body shape, and opacity, as described previously (Meng et al., 2015, 2017a; Xu et al., 2025).

Spatial connection-probability, peak-amplitude, and charge-transfer maps were generated separately for excitatory and inhibitory responses. For each recorded neuron, photostimulation coordinates were transformed into a common soma-centered orientation. At each stimulation site, the precomputed mean peak amplitude and transferred charge of detected postsynaptic responses were assigned to the corresponding spatial location; locations without a detected response were retained as missing values rather than assigned a value of zero. Binary connection maps were generated separately, with responsive sites assigned a value of 1 and nonresponsive sites assigned a value of 0.

Soma-aligned stimulation locations from all cells within an experimental group were pooled and averaged within 40 × 40 µm spatial bins. The resulting connection-probability value represents the proportion of observations with a detected response at each location, whereas the peak-amplitude and charge-transfer values represent the mean magnitude of detected responses contributing to that location. Peak and charge values were displayed only at locations with a group connection probability >10%. The noninterpolated binned maps were retained for quantitative processing, whereas cubic interpolation onto a 1 µm grid was used only to produce the displayed heat maps. NR and DE maps were displayed using identical color limits derived from the pooled noninterpolated group maps.

Layer-specific LSPS measurements were calculated separately for each recorded neuron from the original, noninterpolated cell-level maps. Input area was calculated as the number of responsive stimulation sites within each source layer multiplied by the cortical area represented by each stimulation site. Total charge transfer and total peak input were calculated by summing the corresponding response measurements across responsive locations within each layer. Mean charge transfer and mean peak amplitude were calculated by averaging the respective response measurements across responsive stimulation sites within each layer.

Integration distance was used to quantify the rostrocaudal extent of input within each source layer. Responses were first collapsed across the cortical depth dimension to produce a rostrocaudal input profile. The integration distance was then defined as the rostrocaudal span containing 80% of the cumulative input magnitude. Thus, this measurement reflects the overall rostrocaudal extent of the input distribution rather than the number of responsive sites.

Excitatory–inhibitory balance was calculated separately for each recorded neuron and source layer using matched excitatory and inhibitory measurements from the same cell. The analysis used a normalized E/I index:

$$E/I\;\mathrm{index}=\frac{E-I}{E+I}.$$

Separate E/I indices were calculated for input area, total transferred charge, and total peak input using the corresponding excitatory and inhibitory layer-specific measurements. Values range from −1 to 1, with positive values indicating a relative bias toward excitation, negative values indicating a relative bias toward inhibition, and values near zero indicating a similar relative contribution of excitation and inhibition. An E/I index was not calculated when both the excitatory and inhibitory measurements were zero. The E/I indices were calculated from cell-level measurements and were not obtained by dividing or otherwise combining group means.

To characterize site-specific variability across the population of recorded neurons, Fano-factor maps were calculated separately for each experimental group. At each soma-aligned stimulation location, the Fano factor was calculated across cells as follows:

$$F=\frac{\sigma^{2}}{\mu },$$

where $\sigma^{2}$and μ are the variance and mean, respectively, of the evoked input values at that location. Fano factors were not calculated at locations where the group connection probability was below 10%; these locations are indicated in gray in the corresponding maps. Lower Fano-factor values indicate that input from a given location was relatively consistent across neurons, whereas higher values indicate greater cell-to-cell variability. Difference maps were generated by subtracting the NR and DE Fano-factor maps in the direction specified in the corresponding figure legend.

The similarity of spatial connection patterns among neurons recorded from the same animal was assessed separately for total excitatory and total inhibitory inputs. Each neuron's soma-aligned binary connection-occurrence map contained a value of 1 at stimulation sites where a synaptic response was detected and 0 at sites without a detected response. Maps were restricted to the common analyzed spatial region and converted from two-dimensional grids into one-dimensional vectors while preserving the spatial correspondence among stimulation locations. Within each animal, Pearson’s correlation coefficients were calculated between every unique pair of eligible neuronal maps. A correlation could be calculated only when both maps contained sufficient variation; maps with constant values, for which Pearson’s correlation is undefined, were excluded from that endpoint. For every animal containing at least two eligible neurons, Pearson’s correlations were calculated for every unique, unordered within-animal cell pair. The primary animal-level similarity measure was the arithmetic mean of all valid within-animal Pearson’s coefficients. This produced one similarity value per animal for each input type. Exact correlations of *r* = ±1 were retained in the primary analysis. Higher values indicate that neurons recorded from the same animal had more similar spatial input patterns, values near zero indicate weak spatial correspondence, and negative values indicate inversely organized spatial patterns. Animals represented by only one eligible neuron could not provide a within-animal correlation and therefore did not contribute to that endpoint.

### Statistical analysis

Statistical analyses were performed using MATLAB, GraphPad Prism, and R. Because different numbers of neurons were recorded from individual animals, cell-level, layer-specific LSPS measurements were analyzed using mixed-effects models. Experimental condition (NR or DE) was included as a fixed effect, and animal identity was included as a random effect:

LSPSmetric∼RearingCondition+(1∣AnimalID).

This model accounts for across-animal variability and for the nonindependence of neurons recorded from the same animal, allowing statistical comparison of different numbers of recorded cells from individual animals. Models were fit separately for each LSPS metric and source layer.

For each metric-by-layer comparison, the distributions of the cell-level values were assessed for normality using the Lilliefors test. When both experimental groups satisfied the normality criterion, the effect of experimental condition was evaluated using a linear mixed-effects model. When either group failed the normality criterion, the data were analyzed using an aligned-rank-transform mixed-effects model implemented with the ARTool package, version 2.1.2, in R (Wobbrock et al., 2011). The aligned-rank-transform models retained experimental condition as a fixed effect and animal identity as a random effect. Cumulative distribution functions and box-and-whisker plots were used to visualize the distributions and central tendencies of the cell-level measurements, respectively. Statistical conclusions for layer-specific connection probability, input area, integration distance, charge transfer, peak amplitude, and E/I index were based on the mixed-effects analyses rather than on tests treating individual neurons as independent biological replicates.

Group differences in circuit similarity were evaluated separately for each postsynaptic current type using aligned rank transform models. Every unique within-animal cell pair was calculated once, and the valid Pearson’s coefficients for the total excitatory or inhibitory map were averaged within the animal. The resulting dataset contained one response value per eligible animal for each input type. Because animal identity was already incorporated through this aggregation, no additional AnimalID random effect was required. Group differences were evaluated using aligned rank transform models of the following form:

AnimalmeanPearsonr∼Rearingcondition.

Circuit similarity analysis focused on the similarity of the total excitatory and inhibitory connection-occurrence maps rather than testing similarity separately within each cortical layer. This analysis addressed whether DE altered the overall spatial organization of inputs received by L6-CTN neurons within an animal, integrating information across both cortical depth and lateral position. It therefore complements the layer-specific analyses in Figures 2–6, which tested the area, strength, and lateral extent of input originating from anatomically defined layers.

## Results

To determine whether brief visual deprivation alters intracortical synaptic input to A1 layer 6 corticothalamic neurons (L6-CTNs), we performed whole-cell patch-clamp recordings paired with LSPS of caged glutamate in acute auditory thalamocortical slices. Mice were housed either under normal 12 h light/dark cycle conditions (normally reared, NR; control) or were dark-exposed (DE) for 7 d (initiated from P38 to P58) before recording (Fig. 1*A*). L6-CTNs were identified by Cre-dependent tdTomato expression in Ntsr1-Cre_GN220 × Ai14 mice and targeted under combined infrared and epifluorescence illumination (Fig. 1*B*). Across groups, recorded neurons were sampled from comparable positions within L6 (Fig. 1*C*). For each cell, LSPS was conducted over a predefined grid spanning ∼1 × 1 mm of cortex with 40 µm spacing (Fig. 1*B*). At holding potentials of −70 and 0 mV, we recorded laser uncaging evoked EPSCs and IPSCs, respectively (Fig. 1*D*). Stimulation sites producing large-amplitude inward currents with latencies <8 ms were classified as direct responses and excluded, and only synaptic events within the 8–50 ms window were analyzed. Individual maps were aligned to the soma coordinates, enabling the generation of population-averaged maps for connection probability and synaptic strength (Fig. 1*E*).

**Figure 1.**
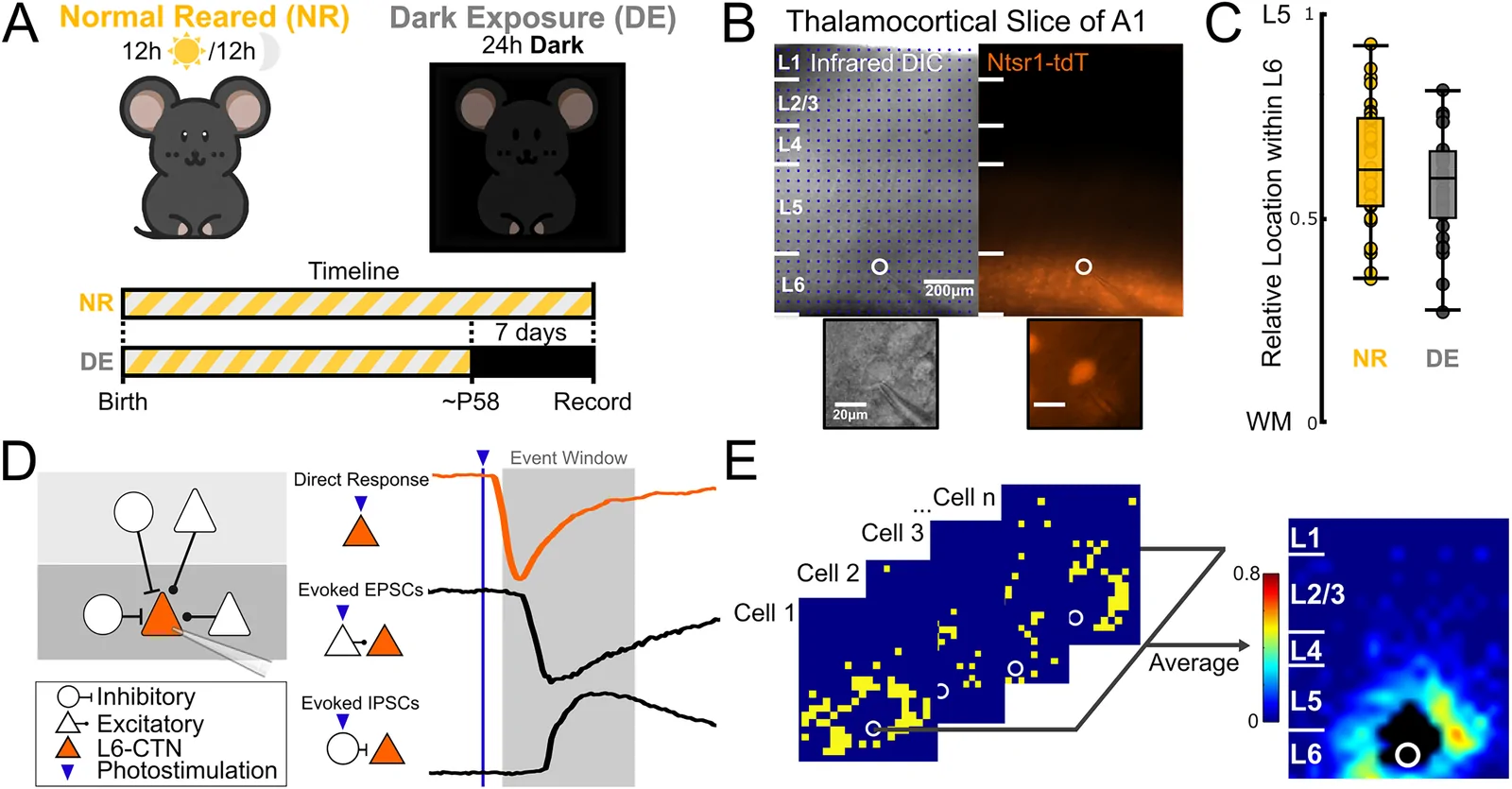
Using LSPS to map excitatory and inhibitory inputs to A1 L6-CTNs. ***A***, Mice were group housed and normally reared (NR) or dark exposed (DE) 1 week prior to dissection and recording. ***B***, Left, Infrared image of a thalamocortical A1 slice with a patch pipette recording from a L6-CTN. Blue dots indicate the laser stimulation grid. White circles mark the soma location of the recorded cell. Below the slice is a zoomed-in infrared image of the patched cell at 40× magnification. Right, Fluorescence imaging of the same slice and cell to visualize Ntsr1-tdT-positive L6-CTNs. ***C***, Normalized laminar positions of recorded L6-CTNs. For each cell, the soma position was expressed as the distance from the soma to the L6/white matter boundary divided by the total distance between the L5/L6 and L6/white-matter boundaries. Thus, 0 corresponds to the L6/white-matter boundary, 0.5 to the midpoint of L6, and 1 to the L5/L6 boundary. Laminar boundaries were identified manually using Ntsr1-tdTomato expression and slice morphology. Boxes span the interquartile range, horizontal lines indicate medians, whiskers extend to the most extreme values within 1.5 times the interquartile range, and circles represent individual recorded cells. No significant effect of rearing condition on normalized soma position was detected using an Aligned Rank Transform mixed-effects model with animal identity included as a random intercept (*F*_(1,34.78)_ = 2.44, *p* = 0.127). ***D***, Left, Schematic of how evoked excitatory and inhibitory inputs are recorded from patched L6-CTNs (orange triangle). Whole-cell voltage-clamp recordings at holding potentials of −70 or 0 mV enable recording of evoked synaptic EPSCs and evoked IPSCs, respectively, through the uncaging of glutamate near an excitatory cell (white triangle) or an inhibitory cell (white circle). Right, Example traces recorded from L6-CTNs following photostimulation (blue arrow and line). Evoked traces that occur within the event window (gray rectangle spanning >8 and <50 ms) were analyzed. Uncaging glutamate on the soma or proximal dendrites of the patched L6-CTN results in a direct response (orange trace) with an onset latency <8 ms. Traces that occur outside of the event window are discarded from the average analysis. ***E***, Left, Schematic illustration of how average maps are produced for qualitative analysis. Individual maps from recorded neurons are aligned to the soma location and averaged to create a single, representative map for a given condition. Right, An example of an averaged map: the averaged map of connection probability for excitatory connections in NR animals. Soma location is indicated by the white circle. White horizontal lines indicate averaged laminar borders and are 100 µm long. Black pixels indicate stimulation sites classified as direct responses.

### Brief visual deprivation reduces excitatory input area from superficial and granular layers onto A1 L6-CTNs

Following dark exposure, A1 undergoes cross-modal adaptation in which feedforward thalamocortical transmission is potentiated (Petrus et al., 2014, 2015) while intracortical excitatory connectivity to L2/3 and L4 is spatially refined and weakened (Meng et al., 2015, 2017a), shifting the circuit toward more reliable bottom-up coding and improved discrimination (Lee and Whitt, 2015). Thus, we expected to observe similar refinement of intracortical connections onto L6-CTNs following 7 d of DE. Individual input maps showed considerable variation from cell to cell, despite recording from comparable populations of L6-CTNs (Fig. 1*E*). To assess overall patterns across the population, we aligned individual maps to the recorded soma position and averaged the connection probability at each stimulation site (Fig. 1*E*). In NR mice, excitatory inputs arose throughout L2/3–L6, with prominent input from L4, L5, and L6 (Fig. 2*A*). The white horizontal lines indicate averaged laminar borders and are 100 µm long. Qualitatively, the DE average map showed reduced connection occurrence in several layers, most notably superficial and granular layers.

**Figure 2.**
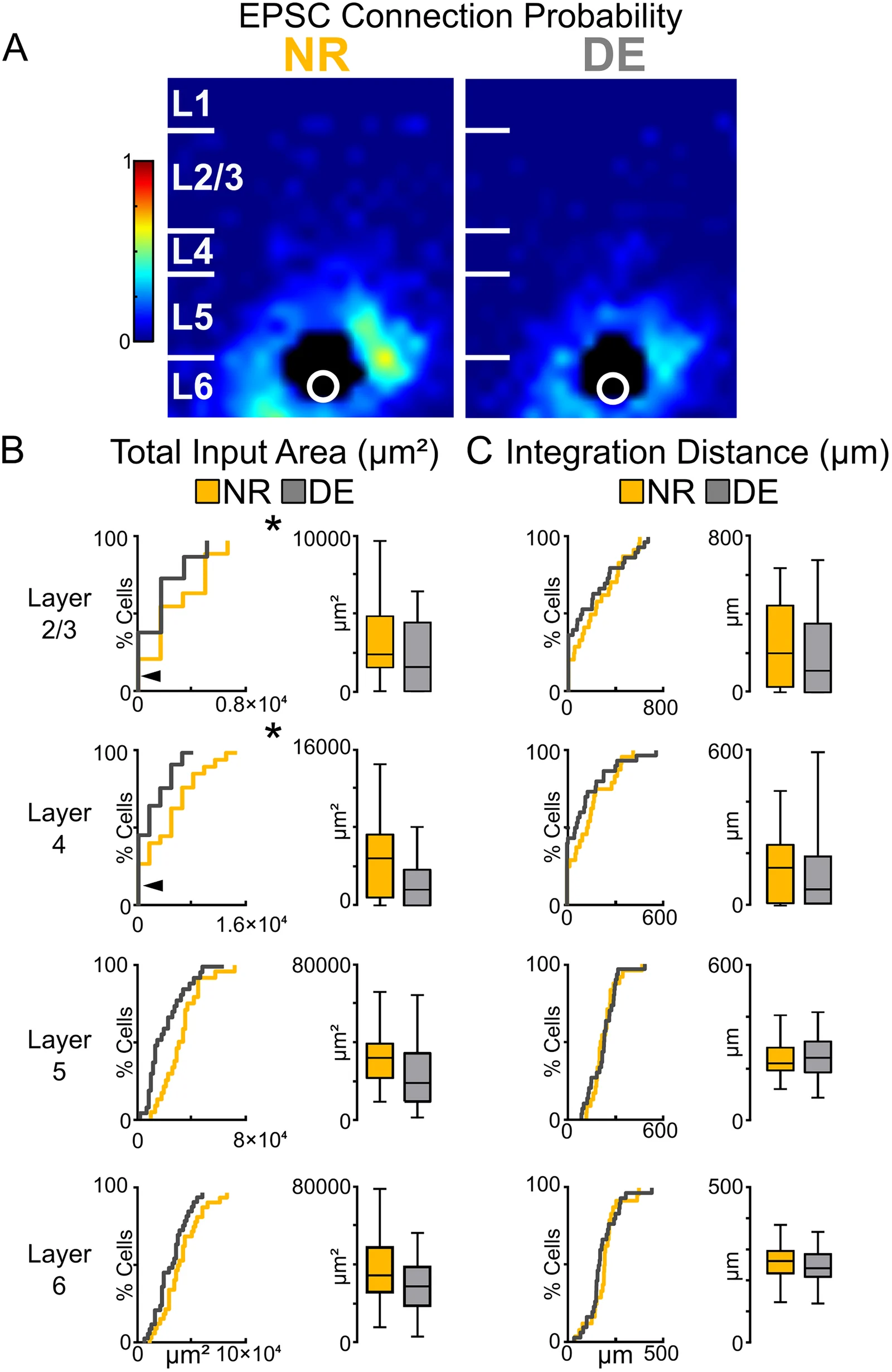
DE reduces excitatory connection probability and input area from superficial and granular layers onto A1 L6-CTNs. ***A***, Heat maps displaying the average excitatory connection probabilities aligned to the soma for NR (yellow; *n* = 32 cells from 19 mice) and DE (gray; *n* = 33 cells from 19 mice) animals. Connection probability is displayed in pseudocolor. The white circle indicates the position of the recorded soma. White horizontal lines indicate averaged laminar boundaries and serve as scale bars (width, 100 µm). Black pixels indicate direct responses. All assessments of excitatory inputs were measured by holding L6-CTNs at −70 mV, the reversal potential for GABA_A_ receptor-mediated currents. ***B***, Layer-specific total excitatory input area onto A1 L6-CTNs. Left, Cumulative distribution functions (CDFs) of total excitatory input area for NR (yellow) and DE (gray) cells. Right, Box-and-whisker plots showing the corresponding cell-level distributions. Boxes indicate the interquartile range (25th–75th percentiles), horizontal lines indicate medians, and whiskers extend to the most extreme values within 1.5 times the interquartile range. Total input area was calculated as the number of stimulation sites that evoked an EPSC multiplied by the area represented by each site (40 × 40 µm; 1,600 µm^2^). Group differences were evaluated separately within each layer using aligned rank transform mixed-effects models, with rearing condition as a fixed effect and animal identity as a random intercept. DE significantly reduced total excitatory input area arising from L2/3 (*F*_(1,30.08)_ = 4.52, *p* = 0.0419) and L4 (*F*_(1,31.99)_ = 6.38, *p* = 0.0167), but not from L5 (*F*_(1,35.33)_ = 3.02, *p* = 0.0910) or L6 (*F*_(1,27.55)_ = 3.48, *p* = 0.0728). Median (IQR 25–75%) excitatory input area for each layer were as follows: NR L2/3 Median 2,400 µm^2^ (IQR 1,600–4,800); DE L2/3 Median 1,600 µm^2^ (IQR 0–3,200), NR L4 Median 4,800 µm^2^ (IQR 800–7,200); DE L4 Median 1,600 µm^2^ (IQR 0–4,800), NR L5 Median 32,000 µm^2^ (IQR 21,600–39,200); DE L5 Median 19,200 µm^2^ (IQR 9,600–34,400), NR L6 Median 34,400 µm^2^ (IQR 25,600–48,800); DE L6 Median 28,800 µm^2^ (IQR 18,800–38,800). ***C***, Layer-specific lateral extent of excitatory input, defined as the rostrocaudal distance encompassing 80% of the total excitatory input within each layer (Dist80). Left, CDFs for NR and DE cells. Right, Box-and-whisker plots of the corresponding cell-level distributions. No significant effects of rearing condition were detected using layer-specific aligned rank transform mixed-effects models with animal identity included as a random intercept: L2/3 *F*_(1,27.55)_ = 1.24, *p* = 0.275; L4 *F*_(1,31.11)_ = 3.50, *p* = 0.0708; L5 *F*_(1,31.41)_ = 0.19, *p* = 0.670; L6 *F*_(1,34.79)_ = 0.396, *p* = 0.533. *p < 0.05.

While these averaged maps provide a useful overview of population-level input patterns and their changes, they do not reflect the differences between individual cells. To quantify excitatory connectivity across neurons, we measured the total area of stimulation sites within each layer that elicited functional inputs in the recorded L6 neuron, which was computed as the number of sites evoking synaptic responses multiplied by the area per grid location, and compared their distributions between groups. Layer-specific aligned rank transform (ART) mixed-effects models, with rearing condition as a fixed effect and animal identity as a random effect, showed that DE reduced excitatory input area arising from L2/3 (*F*_(1,30.08)_ = 4.52, *p* = 0.0419) and L4 (*F*_(1,31.99)_ = 6.38, *p* = 0.0167; Fig. 2*B*). In contrast, group effects did not reach significance for L5 (*F*_(1,35.33)_ = 3.02, *p* = 0.0910) or L6 (*F*_(1,27.55)_ = 3.48, *p* = 0.0728). While each recorded cell in both experimental conditions received excitatory input from L5 and L6, a fraction of cells under NR conditions were devoid of any input from L2/3 and L4. As visible in the CDF, following DE, a larger fraction of cells lacked detectable excitatory input from L2/3 and L4 (black arrow). Thus, the final mixed-effects analysis supports reduced excitatory input area from superficial and granular layers, but not a statistically reliable decrease from L5 or L6.

The observed decrease in the total area that provides functional excitatory input to L6-CTN could be due to a reduction in the density of functional connections or narrowing of the lateral spread of functional inputs from each layer. To assess the lateral spatial extent of locations along the tonotopic axis within each layer that provide functional inputs to L6-CTN, we computed the laminar distance that encompassed 80% of all connected sites within each layer and plotted their cumulative distributions (Fig. 2*C*). The distance required to accumulate 80% of total excitatory input did not differ between NR and DE, indicating that the radial extent of functional connections was unchanged. Together, these results suggest that visual deprivation produces a spatially homogeneous reduction in excitatory connectivity, spanning the same lateral extent, rather than a preferential loss of distal inputs along the tonotopic axis.

### Brief visual deprivation reduces the strength of excitatory input from infragranular layers onto L6-CTNs

We next evaluated whether changes in connection probability were accompanied by alterations in synaptic strength (Fig. 3). Soma-aligned average maps showed the spatial distributions of EPSC charge transfer and peak amplitude using shared NR–DE pseudocolor scales (Fig. 3*A*,*B*). At each eligible stimulation location, averages were calculated only when responses were detected in >10% of cells. These maps qualitatively summarize spatial response magnitude. For each cell receiving input from a given layer, mean EPSC charge transfer was calculated across detected responses. Layer-specific ART mixed-effects models showed that DE reduced mean excitatory charge transfer from L5 (*F*_(1,32.85)_ = 4.15, *p* = 0.0498), but not from L2/3 (*F*_(1,25.75)_ = 1.24, *p* = 0.276), L4 (*F*_(1,20.67)_ = 1.01, *p* = 0.325), or L6 (*F*_(1,28.25)_ = 1.92, *p* = 0.176; Fig. 3*C*). Mean EPSC peak amplitude was also reduced for inputs arising from L5 (*F*_(1,32.60)_ = 7.96, *p* = 0.00807) and L6 (*F*_(1,27.55)_ = 7.77, *p* = 0.00951), with no significant group effect for L2/3 (*F*_(1,25.75)_ = 0.16, *p* = 0.696) or L4 (*F*_(1,18.15)_ = 2.25, *p* = 0.151; Fig. 3*D*). Taken together, these analyses suggest that DE reduces the density of excitatory interlaminar inputs from L2 through L4 onto L6-CTNs, while also reducing the average strength of excitatory connections originating from infragranular layers.

**Figure 3.**
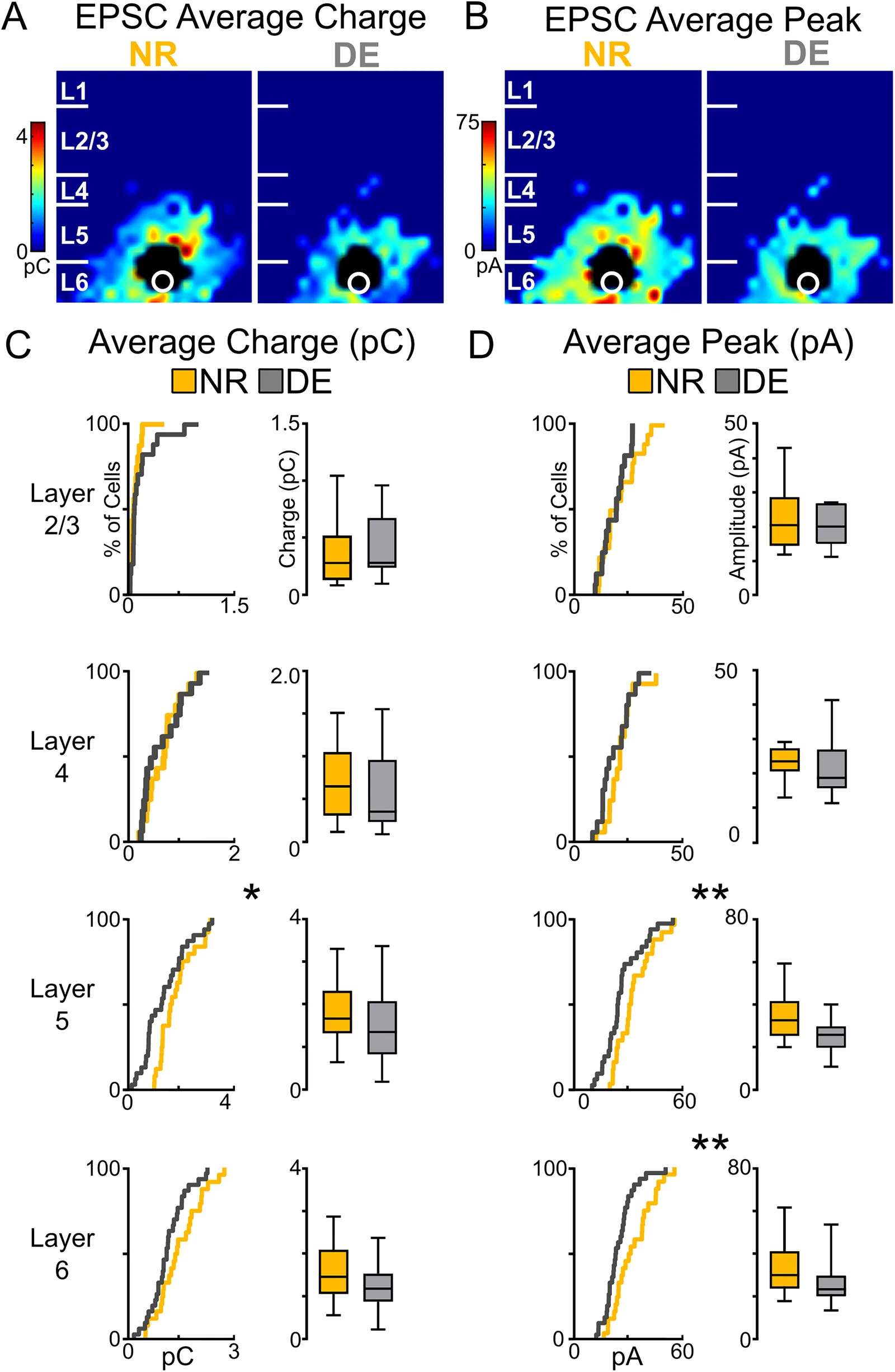
DE produces layer-specific reductions in the strength of excitatory input onto A1 L6-CTNs. ***A***, Soma-aligned heat maps displaying the average charge transfer of excitatory postsynaptic responses in NR (left; *n* = 32 cells from 19 mice) and DE (right; *n* = 33 cells from 19 mice) animals. Average EPSC charge is displayed in pseudocolor. At each stimulation location, averages were calculated only when responses were detected in >10% of cells. The white circle indicates the position of the recorded soma. White horizontal lines indicate average laminar boundaries and provide a spatial scale (horizontal extent, 100 µm). Black pixels indicate stimulation sites classified as direct responses. NR and DE charge maps were displayed using a shared pseudocolor scale of 0–4.7 pC. The upper limit was derived by pooling the eligible spatial-bin values across both conditions and rounding the pooled maximum (4.685 pC) to 4.7 pC; no bins exceeded this limit. ***B***, Soma-aligned heat maps displaying the average peak amplitude of excitatory postsynaptic responses in NR (left; *n* = 32 cells from 19 mice) and DE (right; *n* = 33 cells from 19 mice) animals. Average EPSC peak amplitude is displayed in pseudocolor. At each stimulation location, averages were calculated only when responses were detected in >10% of cells. White horizontal lines indicate averaged laminar boundaries (width, 100 µm). Black pixels indicate stimulation sites classified as direct responses. NR and DE peak-amplitude maps were displayed using a shared pseudocolor scale of 0–75 pA. The upper limit was derived by pooling the eligible spatial-bin values across both conditions and rounding the pooled maximum (74.69 pA) to 75 pA; no eligible bins exceeded this limit. ***C***, Layer-specific mean charge transfer of excitatory inputs onto A1 L6-CTNs. Left, Cumulative distribution functions (CDFs) for NR (yellow) and DE (gray) cells. Right, Box-and-whisker plots showing the corresponding cell-level distributions. Boxes span the interquartile range (IQR; 25th–75th percentiles), horizontal lines indicate medians, and whiskers extend to the most extreme values within 1.5 times the IQR. Averages were calculated from detected EPSCs for cells that received >0 input from a given layer (L2/3 NR *n* = 25 cells from 17 mice, DE *n* = 21 cells from 15 mice; L4 NR *n* = 24 cells from 15 mice, DE *n* = 17 cells from 11 mice; L5 NR *n* = 32 cells from 19 mice, DE *n* = 33 cells from 19 mice; L6 NR *n* = 32 cells from 19 mice, DE *n* = 33 cells from 19 mice). Group differences were evaluated separately within each layer using Aligned Rank Transform mixed-effects models, with rearing condition as a fixed effect and animal identity as a random intercept. DE significantly reduced mean excitatory charge transfer from L5 (*F*_(1,32.85)_ = 4.15, *p* = 0.0498), but not from L2/3 (*F*_(1,25.75)_ = 1.24, *p* = 0.276), L4 (*F*_(1,20.67)_ = 1.01, *p* = 0.325), or L6 (*F*_(1,28.25)_ = 1.92, *p* = 0.176). Median (IQR) charge transfer in NR and DE cells, respectively, was 1.66 (1.34–2.29) and 1.35 (0.86–2.05) pC for L5 input. ***D***, Layer-specific mean peak amplitude of excitatory inputs onto A1 L6-CTNs. Averages were calculated from detected EPSCs for cells that received >0 input from a given layer. Left, CDFs for NR (yellow) and DE (gray) cells. Right, Box-and-whisker plots showing the corresponding cell-level distributions. Cell numbers and boxplot conventions are as described in ***C***. Layer-specific Aligned Rank Transform mixed-effects models showed that DE significantly reduced mean EPSC peak amplitude arising from L5 (*F*_(1,32.60)_ = 7.96, *p* = 0.0081) and L6 (*F*_(1,27.55)_ = 7.77, *p* = 0.0095), but not from L2/3 (*F*_(1,25.75)_ = 0.16, *p* = 0.6963) or L4 (*F*_(1,18.15)_ = 2.25, *p* = 0.1511). Median (IQR) peak amplitudes in NR and DE cells, respectively, were 32.56 (25.72–41.08) and 25.83 (20.23–29.20) pA for L5 input and 30.06 (24.15–40.73) and 23.50 (20.62–29.28) pA for L6 input. *p < 0.05, **p < 0.01.

### Inhibitory input area and lateral extent remain stable following brief visual deprivation

Because excitatory and inhibitory inputs jointly shape the integration properties of L6-CTNs, we next examined whether brief DE similarly affects intracortical inhibition. IPSC input maps were generated at a holding potential of 0 mV (the reversal potential for glutamate receptors, preventing EPSCs) and analyzed using the same response window and direct-response exclusions as the excitatory maps. Inhibitory inputs arose throughout L1–L6 in both groups (Fig. 4*A*). Although the population-average maps showed minor qualitative local differences between NR and DE, layer-specific ART mixed-effects models detected no significant effect of rearing condition on inhibitory input area from L1–3 (*F*_(1,33.73)_ = 1.39, *p* = 0.247), L4 (*F*_(1,35.15)_ = 1.22, *p* = 0.277), L5 (*F*_(1,34.14)_ = 2.34, *p* = 0.136), or L6 (*F*_(1,35.44)_ = 0.63, *p* = 0.431; Fig. 4*B*).

**Figure 4.**
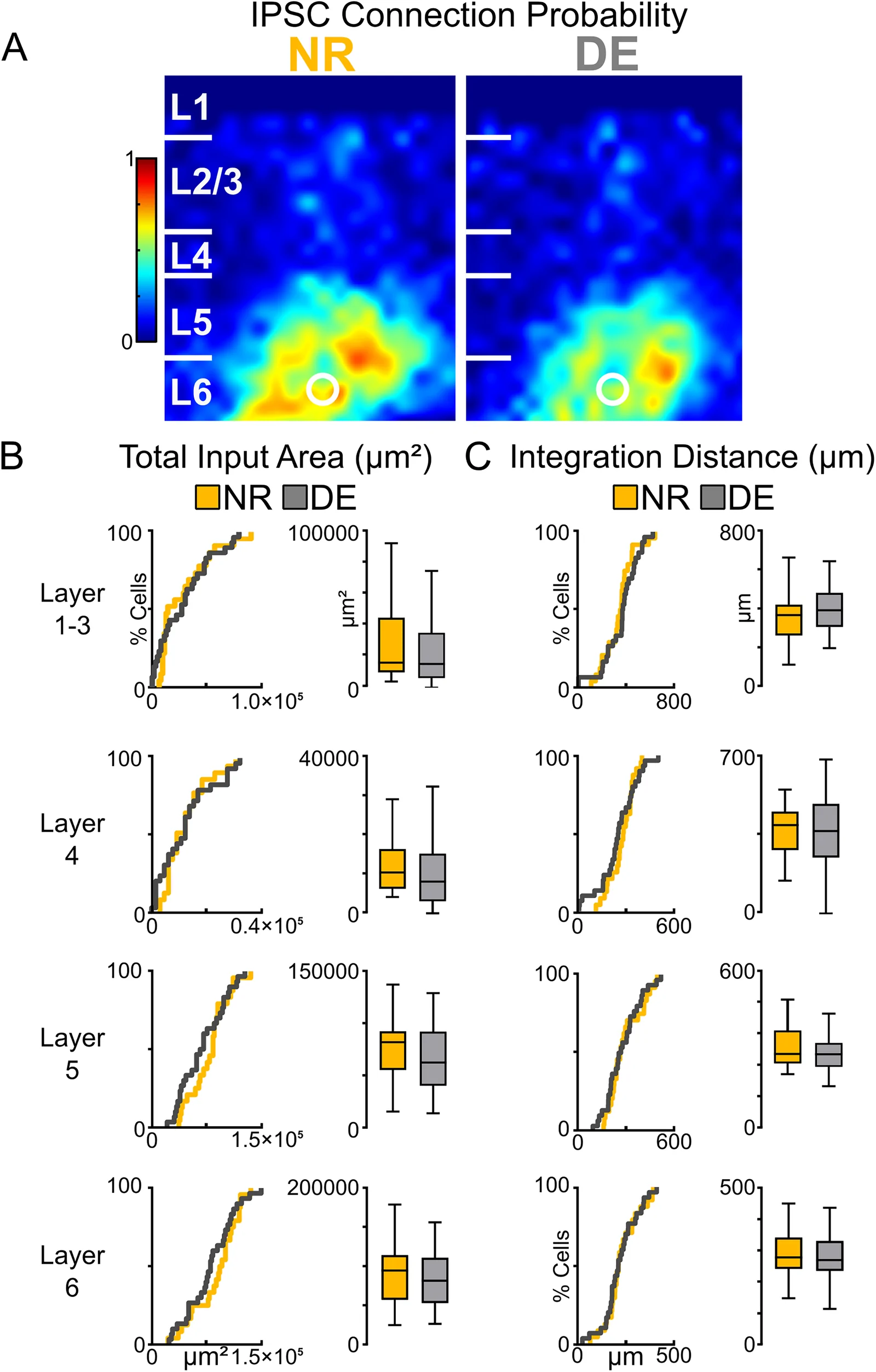
DE does not significantly alter inhibitory input area or spatial extent onto A1 L6-CTNs. ***A***, Soma-aligned heat maps displaying average inhibitory connection probability for NR (yellow; *n* = 32 cells from 19 mice) and DE (gray; *n* = 33 cells from 19 mice) animals. Connection probability is displayed in pseudocolor, and the white circle indicates the position of the recorded soma. White horizontal lines indicate average laminar boundaries and provide a spatial scale (horizontal extent, 100 µm). Black pixels indicate stimulation sites excluded because they were classified as direct responses. Inhibitory inputs were measured by holding L6-CTNs at 0 mV, near the reversal potential for glutamatergic receptor-mediated currents. ***B***, Layer-specific total inhibitory input area onto A1 L6-CTNs. Left, Cumulative distribution functions (CDFs) of total inhibitory input area for NR (yellow) and DE (gray) cells. Right, Box-and-whisker plots showing the corresponding cell-level distributions. Boxes span the interquartile range (IQR; 25th–75th percentiles), horizontal lines indicate medians, and whiskers extend to the most extreme values within 1.5 times the IQR. Total input area was calculated as the number of stimulation sites that evoked an IPSC multiplied by the area represented by each site (40 × 40 µm; 1,600 µm^2^). Group differences were evaluated separately within each layer using Aligned Rank Transform mixed-effects models, with rearing condition as a fixed effect and animal identity as a random intercept. No significant effect of rearing condition was detected for input arising from L1–3 (*F*_(1,33.73)_ = 1.39, *p* = 0.247), L4 (*F*_(1,35.15)_ = 1.22, *p* = 0.277), L5 (*F*_(1,34.14)_ = 2.34, *p* = 0.136), or L6 (*F*_(1,35.44)_ = 0.63, *p* = 0.431). Median (IQR) input areas for NR and DE cells, respectively, were as follows: L1–3, 15,200 (9,600–43,200) and 14,400 (6,000–33,600) µm^2^; L4, 10,400 (6,400–16,000) and 8,000 (3,200–17,600) µm^2^; L5, 81,600 (56,000–91,200) and 62,400 (41,200–90,800) µm^2^; and L6, 94,400 (58,400–112,800) and 81,600 (54,400–109,600) µm^2^. ***C***, Layer-specific lateral extent of inhibitory input, defined as the rostrocaudal distance encompassing 80% of the total inhibitory input within each layer (Dist80). Left, CDFs for NR (yellow) and DE (gray) cells. Right, Box-and-whisker plots showing the corresponding cell-level distributions. No significant effects of rearing condition were detected using layer-specific Aligned Rank Transform mixed-effects models with animal identity included as a random intercept: L1–3 (*F*_(1,27.55)_ = 1.99, *p* = 0.170), L4 (*F*_(1,28.46)_ = 0.05, *p* = 0.826), L5 (*F*_(1,31.10)_ = 0.37, *p* = 0.548), and L6 (*F*_(1,33.49)_ = 0.310, *p* = 0.581).

The distance required to accumulate 80% of total inhibitory input was likewise unchanged for L1–3 (*F*_(1,27.55)_ = 1.99, *p* = 0.170), L4 (*F*_(1,28.46)_ = 0.05, *p* = 0.826), L5 (*F*_(1,31.10)_ = 0.37, *p* = 0.548), and L6 (*F*_(1,33.49)_ = 0.31, *p* = 0.581; Fig. 4*C*). Thus, DE did not produce a detectable change in either the total area or lateral extent of inhibitory inputs onto L6-CTNs.

### Intracortical inhibitory input strength onto A1 L6-CTNs remains stable following brief visual deprivation

We next quantified inhibitory synaptic strength by comparing the average inhibitory charge transfer and average peak amplitude of IPSCs onto L6-CTNs from each layer (Fig. 5). ART mixed-effects models detected no significant effect of DE on mean inhibitory charge transfer from L1–3 (*F*_(1,31.71)_ = 0.84, *p* = 0.368), L4 (*F*_(1,29.67)_ = 0.44, *p* = 0.511), L5 (*F*_(1,31.30)_ = 2.32, *p* = 0.138), or L6 (*F*_(1,34.23)_ < 0.01, *p* = 0.993; Fig. 5*C*). Mean inhibitory peak amplitude also did not differ significantly for L1–3 (*F*_(1,31.28)_ = 0.01, *p* = 0.927), L4 (*F*_(1,30.18)_ = 3.19, *p* = 0.0840), L5 (*F*_(1,33.39)_ = 3.13, *p* = 0.0861), or L6 (*F*_(1,34.41)_ = 0.62, *p* = 0.436; Fig. 5*D*). Thus, in contrast to the reductions in excitatory area and deep-layer excitatory strength, the area, lateral extent, and mean strength of inhibitory input remained stable following DE.

**Figure 5.**
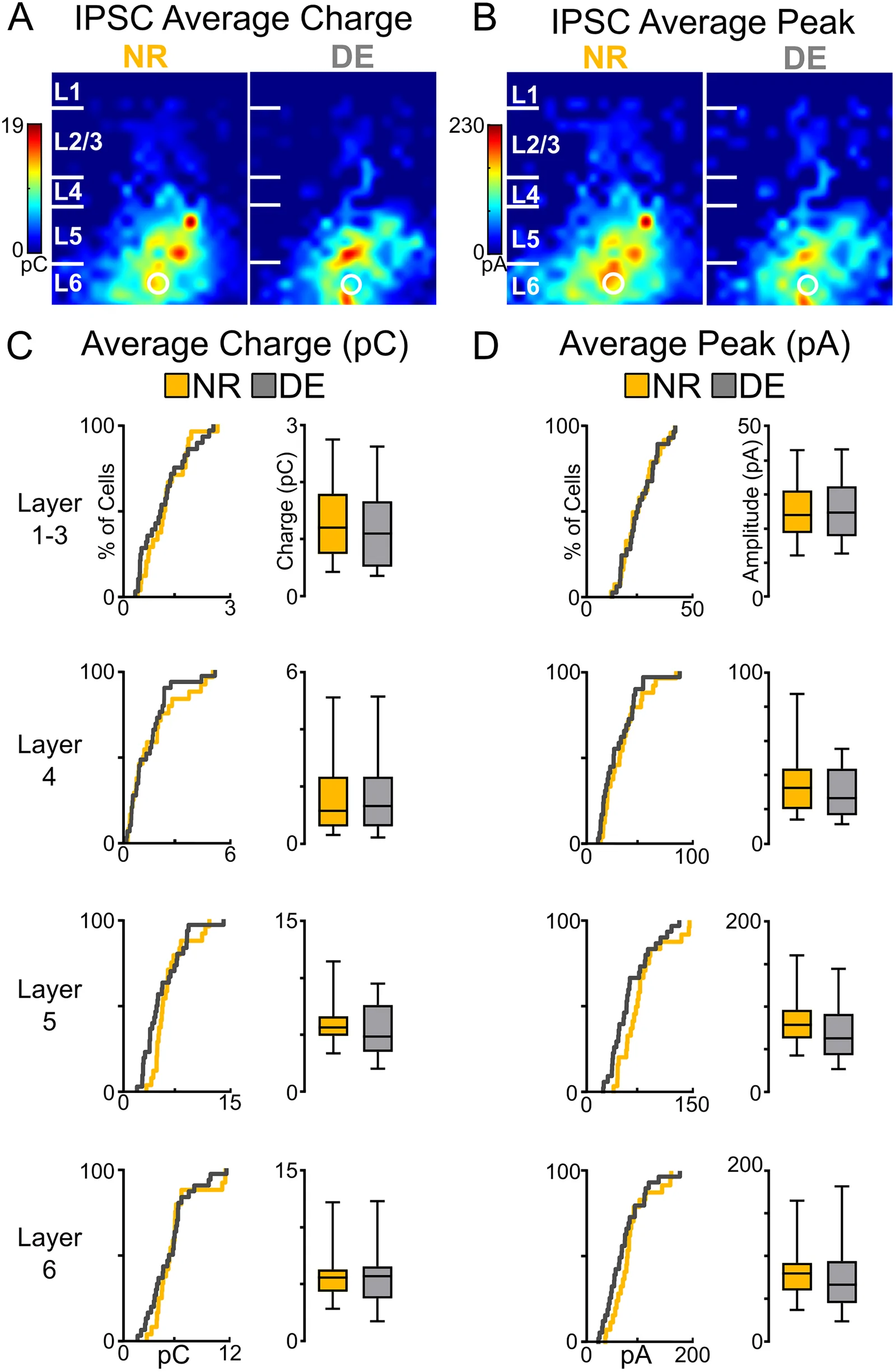
DE does not significantly alter the strength of inhibitory input onto A1 L6-CTNs. ***A***, Soma-aligned heat maps displaying the average charge transfer of inhibitory postsynaptic responses in NR (left; *n* = 32 cells from 19 mice) and DE (right; *n* = 33 cells from 19 mice) animals. Average IPSC charge is displayed in pseudocolor. At each stimulation location, averages were calculated only when responses were detected in >10% of cells. The white circle indicates the position of the recorded soma. White horizontal lines indicate average laminar boundaries and provide a spatial scale (horizontal extent, 100 µm). Black pixels indicate stimulation sites excluded because they were classified as direct responses. NR and DE charge maps were displayed using a shared pseudocolor scale of 0–19 pC. The upper limit was derived by pooling eligible spatial-bin values across both conditions and rounding the pooled maximum (18.64 pC) to 19 pC; no eligible bin values exceeded this limit. ***B***, Soma-aligned heat maps displaying the average peak amplitude of inhibitory postsynaptic responses in NR (left; *n* = 32 cells from 19 mice) and DE (right; *n* = 33 cells from 19 mice) animals. Average IPSC peak amplitude is displayed in pseudocolor. At each stimulation location, averages were calculated only when responses were detected in >10% of cells. Other spatial conventions are as described in ***A***. NR and DE peak-amplitude maps were displayed using a shared pseudocolor scale of 0–230 pA. The upper limit was derived by pooling eligible spatial-bin values across both conditions and rounding the pooled maximum (223.28 pA) to 230 pA; no eligible bin values exceeded this limit. ***C***, Layer-specific mean charge transfer of inhibitory inputs onto A1 L6-CTNs. Means were calculated from detected IPSCs for cells that received input from the indicated layer. Left, Cumulative distribution functions (CDFs) for NR (yellow) and DE (gray) cells. Right, Box-and-whisker plots showing the corresponding cell-level distributions. Boxes span the interquartile range (IQR; 25th–75th percentiles), horizontal lines indicate medians, and whiskers extend to the most extreme values within 1.5 times the IQR. The numbers of cells included were as follows: L1–3, NR *n* = 32 and DE *n* = 31; L4, NR *n* = 31 and DE *n* = 32; L5, NR *n* = 32 and DE *n* = 33; and L6, NR *n* = 32 and DE *n* = 33. Group differences were evaluated separately within each layer using Aligned Rank Transform mixed-effects models, with rearing condition as a fixed effect and animal identity as a random intercept. No significant effect of rearing condition was detected for mean inhibitory charge arising from L1–3 (*F*_(1,31.71)_ = 0.84, *p* = 0.368), L4 (*F*_(1,29.67)_ = 0.44, *p* = 0.511), L5 (*F*_(1,31.30)_ = 2.32, *p* = 0.138), or L6 (*F*_(1,34.23)_ < 0.01, *p* = 0.993). ***D***, Layer-specific mean peak amplitude of inhibitory inputs onto A1 L6-CTNs. Means were calculated from detected IPSCs for cells that received input from the indicated layer. Left, CDFs for NR (yellow) and DE (gray) cells. Right, Box-and-whisker plots showing the corresponding cell-level distributions. Cell numbers and boxplot conventions are as described in ***C***. Layer-specific Aligned Rank Transform mixed-effects models detected no significant effect of rearing condition for mean IPSC peak amplitude arising from L1–3 (*F*_(1,31.28)_ = 0.01, *p* = 0.927), L4 (*F*_(1,30.18)_ = 3.19, *p* = 0.084), L5 (*F*_(1,33.39)_ = 3.13, *p* = 0.086), or L6 (*F*_(1,34.41)_ = 0.62, *p* = 0.436).

### Brief visual deprivation shifts the E/I balance of L4 inputs onto A1 L6-CTNs toward inhibition

Finally, to assess how group differences in excitation and inhibition relate to overall excitatory-to-inhibitory synaptic balance, we calculated cell-level excitatory-to-inhibitory (E/I) indices for input area, charge, and peak amplitude within each layer (Fig. 6). Layer-specific ART mixed-effects models showed that DE reduced the L4 E/I index for input area (*F*_(1,34.15)_ = 5.86, *p* = 0.0210), charge (*F*_(1,33.54)_ = 5.10, *p* = 0.0306), and peak amplitude (*F*_(1,33.20)_ = 4.95, *p* = 0.0330). No significant group effects were detected for any of the three E/I metrics in L1–3, L5, or L6 (all *p* ≥ 0.268). These results identify a layer-selective shift in L4 input toward relatively greater inhibitory influence after DE.

**Figure 6.**
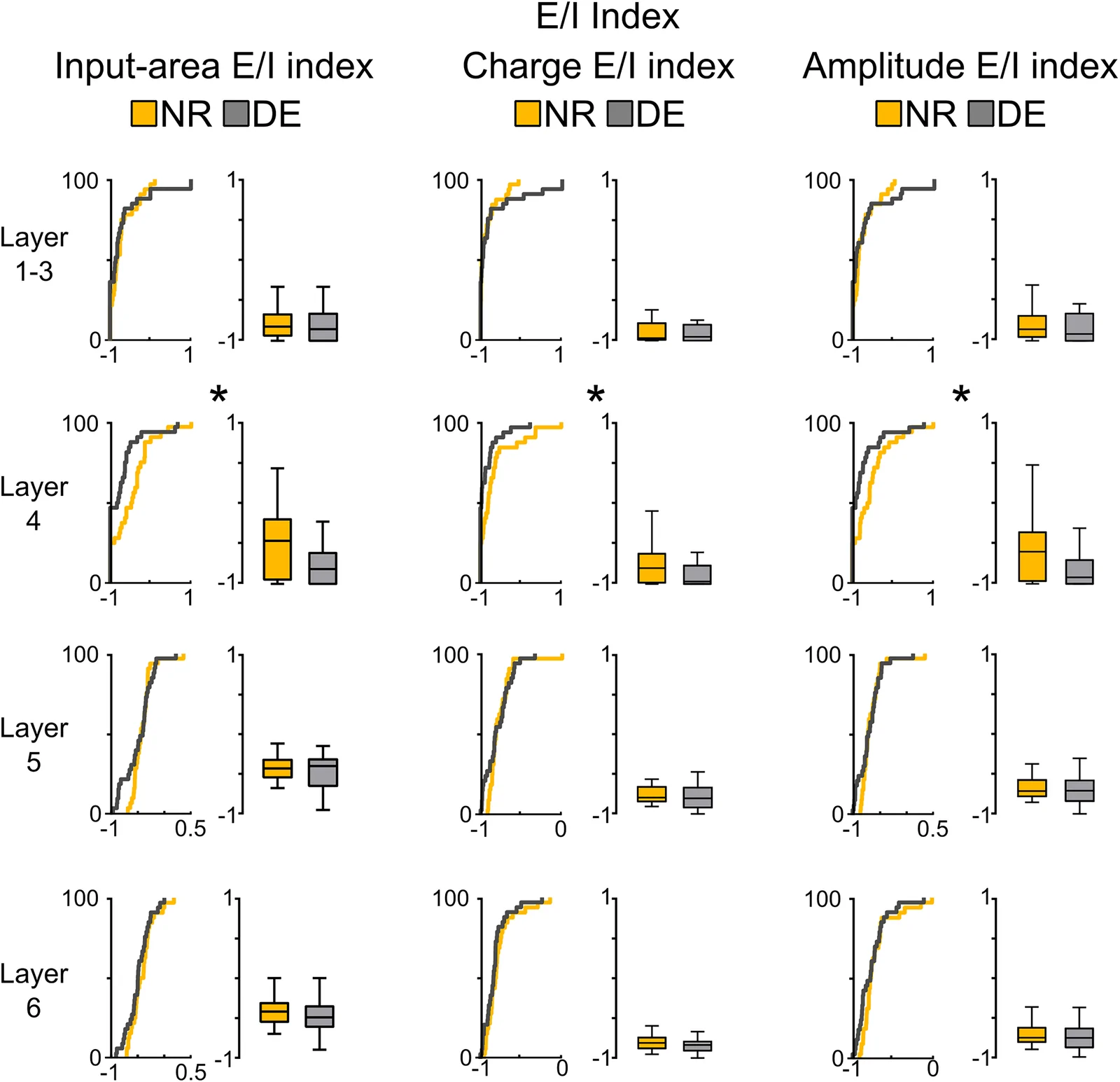
DE shifts the E/I balance of interlaminar inputs from L4 in favor of inhibition. Cell-level cumulative distribution functions (CDFs) and box-and-whisker plots of E/I indices calculated from total input area (left), charge transfer (middle), and peak amplitude (right) for inputs arising from L1–3, L4, L5, and L6. NR data are shown in yellow and DE data in gray. Boxes span the interquartile range (IQR; 25th–75th percentiles), horizontal lines indicate medians, and whiskers extend to the most extreme values within 1.5 times the IQR. E/I indices were calculated separately for input area, charge transfer, and peak amplitude as (*E* − *I*) / (*E* + *I*), where *E* and *I* represent the corresponding nonnegative excitatory and inhibitory input magnitudes. Index values range from −1 to +1, with positive values indicating relatively greater excitation, zero indicating equal excitation and inhibition, and negative values indicating relatively greater inhibition. Group differences were evaluated separately within each layer using Aligned Rank Transform mixed-effects models, with rearing condition as a fixed effect and animal identity as a random intercept. DE significantly reduced the E/I indices of L4 inputs calculated from total input area (*F*_(1,34.15)_ = 5.86, *p* = 0.021), charge transfer (*F*_(1,33.54)_ = 5.10, *p* = 0.0306), and peak amplitude (*F*_(1,33.20)_ = 4.95, *p* = 0.033), indicating a shift toward relatively greater inhibition. Median (IQR) L4 E/I indices for NR and DE cells, respectively, were −0.481 (−0.974 to −0.264) and −0.917 (−1.000 to −0.636) for input area; −0.810 (−0.992 to −0.640) and −1.000 (−1.000 to −0.880) for charge transfer; and −0.628 (−0.983 to −0.395) and −1.000 (−1.000 to −0.801) for peak amplitude.

### Brief visual deprivation alters the spatial organization of excitatory inputs onto A1 L6-CTNs

The preceding analyses showed that DE selectively reduced the area and strength of excitatory inputs onto L6-CTN neurons while leaving the measured area, lateral extent, and strength of inhibitory inputs largely unchanged. However, experience-dependent plasticity can reorganize the spatial distribution of synaptic inputs without necessarily changing overall input strength, reflecting changes in the patterning of intracortical connectivity rather than its magnitude (Feldman, 2009; Meng et al., 2014; Froemke, 2015; Xu et al., 2025). We therefore examined two complementary features of input organization: across-cell variability at individual stimulation locations and within-animal similarity of complete spatial input maps (Fig. 7).

**Figure 7.**
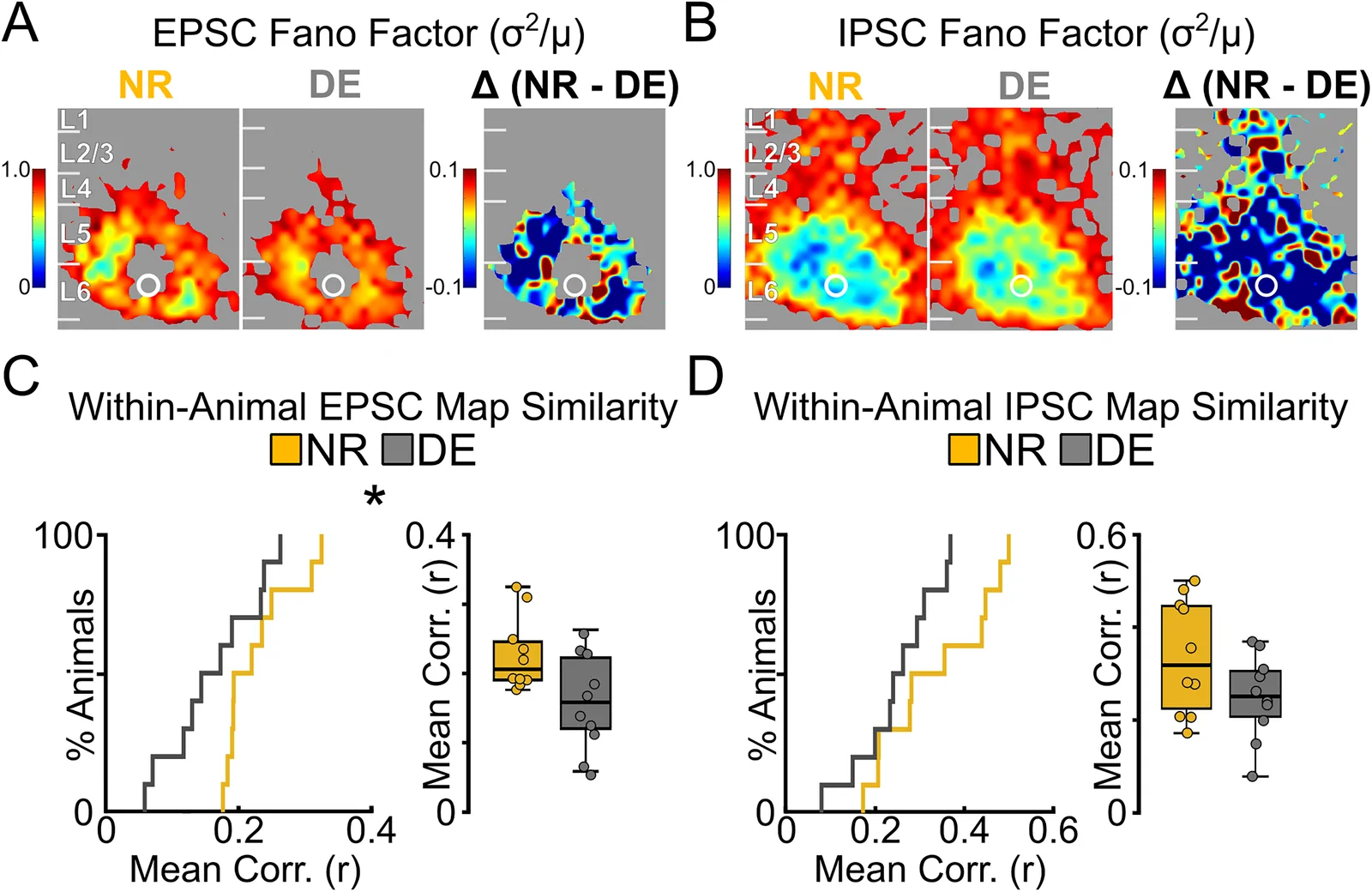
DE selectively reduces within-animal similarity of total EPSC input maps onto A1 L6-CTN neurons. ***A***, Soma-aligned maps displaying the across-cell Fano factor (variance/mean) of excitatory connection-occurrence maps in NR and DE animals, together with the NR − DE difference map. The Fano factor was calculated at each stimulation site from cell-level excitatory response occurrence values and reflects across-cell variability relative to the corresponding mean. Sites with a mean response probability <5% were not evaluated and are displayed in gray. Individual-condition maps are displayed from 0 to 1, and the difference map from −0.1 to 0.1. Maps were interpolated for display. The white circle indicates the recorded soma position, and white horizontal lines indicate average laminar boundaries and provide a spatial scale (horizontal extent, 100 µm). ***B***, Soma-aligned maps displaying the across-cell Fano factor of inhibitory connection-occurrence maps in NR and DE animals, together with the NR − DE difference map. Calculation, display, and spatial conventions are as described in ***A***. ***C***, Within-animal EPSC map similarity. Left, Cumulative distribution functions (CDFs) of animal-level similarity values for NR (yellow) and DE (gray) animals. Right, Box-and-whisker plots showing the corresponding animal-level distributions. For each animal containing at least two eligible neurons, Pearson’s correlations were calculated between every unique pair of soma-aligned binary EPSC connection-occurrence maps. Each cell pair was included once, and the resulting correlations were averaged to produce one similarity value per animal. Higher values indicate more similar spatial input patterns among neurons recorded from the same animal, values near zero indicate weak spatial correspondence, and negative values indicate inversely organized spatial patterns. Animals containing fewer than two eligible neurons were not included. Boxes span the interquartile range (IQR; 25th–75th percentiles), horizontal lines indicate medians, and whiskers extend to the most extreme values within 1.5 times the IQR. The analysis included 22 cells from *N* = 9 NR animals and 24 cells from *N* = 10 DE animals. DE reduced within-animal EPSC map similarity (NR median, 0.193; IQR, 0.190–0.249; DE median, 0.158; IQR, 0.120–0.223; aligned rank transform: *F*_(1,17)_ = 4.61, *p* = 0.0465). ***D***, Within-animal IPSC map similarity. Calculation, eligibility criteria, and plotting conventions are as described in ***C***, using soma-aligned binary IPSC connection-occurrence maps. The analysis included 22 cells from *N* = 9 NR animals and 24 cells from *N* = 10 DE animals. Within-animal IPSC map similarity did not differ significantly between conditions (NR median, 0.281; IQR, 0.207–0.447; DE median, 0.251; IQR, 0.207–0.305; *F*_(1,17)_ = 1.55, *p* = 0.231).

To visualize site-specific cell-to-cell variability, we computed the Fano factor (σ^2^/μ) of LSPS-evoked responses at each stimulation site. Higher Fano-factor values indicate greater cell-to-cell variability in response occurrence at a given stimulation location relative to its mean occurrence probability, whereas lower values indicate more consistent response occurrence across cells. Fano factor maps of EPSCs reveal that excitatory input organization was already characterized by high relative cell-to-cell variability (Fig. 7*A*), whereas inhibitory input occurrence appeared comparatively more consistent in some regions (Fig. 7*B*). Following DE, both maps showed localized increases in relative variability, including a loss of some lower-variability regions in the inhibitory map. Consistent with these results, mapping the differences in Fano factor (Δ; NR − DE) indicated an increase in variability (negative values; cooler color heat map) following DE for both excitatory and inhibitory circuits (Fig. 7*A*,*B*).

We next measured whether neurons recorded from the same animal received inputs from similar spatial locations. For each animal containing at least two eligible neurons, Pearson’s correlations were calculated between every unique pair of soma-aligned binary connection-occurrence maps. Each pair was included once, and the resulting correlation coefficients were averaged to produce one total-map similarity value per animal. Consequently, unlike the across-cell Fano-factor maps, this analysis specifically quantified within-animal map similarity. This approach gave every eligible animal equal weight regardless of the number of neurons or cell pairs it contributed. Higher animal-level mean correlations indicate that neurons from the same animal had more stereotyped spatial input patterns.

DE reduced within-animal similarity of total EPSC connection-occurrence maps (NR: median, 0.193; IQR, 0.190–0.249; DE: median, 0.158; IQR, 0.120–0.223; *F*_(1,17)_ = 4.61, *p* = 0.0465; Fig. 7*C*). Within-animal similarity of total IPSC maps was also numerically lower after DE, but this difference was not statistically significant (NR: median, 0.281; IQR, 0.207–0.447; DE: median, 0.251; IQR, 0.207–0.305; *F*_(1,17)_ = 1.55, *p* = 0.231; Fig. 7*D*).

Together, these analyses indicate that DE selectively reduces the stereotypy of total excitatory connection patterns among L6-CTN neurons recorded from the same animal. This effect was not accompanied by a statistically supported change in total inhibitory map similarity.

## Discussion

Here, we used LSPS to determine how brief adult-onset visual deprivation alters the intracortical synaptic input received by A1 L6 corticothalamic neurons (L6-CTNs). The resulting plasticity was selective rather than a uniform weakening of the local circuit. DE reduced excitatory input area from L2/3 and L4 without detectably changing the lateral extent of those inputs (Fig. 2). Excitatory synaptic strength was altered in deeper layers: L5 input showed reduced mean charge transfer and peak amplitude, whereas recurrent L6 input showed reduced peak amplitude but preserved mean charge transfer (Fig. 3). In contrast, inhibitory input area, lateral extent, mean charge transfer, and peak amplitude were not significantly altered in any layer (Figs. 4, 5). These changes converged selectively in L4, where the E/I ratios for input area, charge, and peak amplitude were shifted toward inhibition, while E/I balance remained stable in L1–3, L5, and L6 (Fig. 6). Collectively, these results indicate that DE can reorganize where synaptic inputs originate in a layer-specific manner across neurons in the primary auditory cortex even when their average spatial area or strength of inputs is preserved. In addition to selective changes in synaptic strength and E/I balance, the total-map analysis further indicated that DE altered how consistently excitatory connections were spatially organized among neurons recorded from the same animal (Fig. 7). Mean within-animal EPSC map similarity was significantly lower after DE. Because these correlations were calculated from binary connection-occurrence maps, the effect could reflect fewer shared connected locations, redistribution of connected sites, or a combination of both. In particular, the reduction in excitatory input area identified in Figure 2 may contribute to the decrease in total-map similarity. The present analysis therefore supports reduced stereotypy of total excitatory connection patterns.

Sensory deprivation induces robust cross-modal plasticity in spared sensory modalities, affecting both feedforward and intracortical circuits. We have previously shown that visual deprivation in adult mice, beyond the auditory thalamocortical critical period, reduces TRN synaptic inhibition onto the MGBv (Whitt et al., 2022), strengthens thalamocortical synaptic connections between the MGBv and A1 L4 (Petrus et al., 2014), and strengthens corticocortical synaptic connections from L4 to L2/3 (Petrus et al., 2015). This visual deprivation-induced configuration favoring feedforward processing would be expected to increase cortical gain and lower sound detection thresholds by amplifying thalamic input to the cortex. Increased feedforward gain is often associated with broader frequency responsiveness, as weaker or nonpreferred inputs become more likely to cross the spiking threshold (Wehr and Zador, 2003; Li et al., 2013). However, visual deprivation has been reported to both lower auditory thresholds and narrow frequency selectivity in A1 (Petrus et al., 2014) as well as increase responsiveness to high-frequency sounds (Solarana et al., 2019). This dissociation suggests that strengthening of feedforward excitatory drive alone is insufficient to explain the perceptual consequences of cross-modal visual deprivation and instead implicates plasticity of intracortical circuits that can refine and selectively enhance auditory function.

Our previous work mapping A1 intracortical circuitry revealed that DE-induced potentiation of feedforward driving inputs corresponds with concomitant intracortical refinement. This refinement manifests as reduced excitatory and inhibitory inputs from L2/3 onto L4 pyramidal neurons, as well as a cortex-wide reduction of excitation and inhibition onto L2/3 neurons (Meng et al., 2015, 2017a). Such laminar-specific refinement is well positioned to enhance frequency selectivity by restricting the spread of intracortical excitation and sharpening stimulus-evoked representations. While refinement of L2/3–L4 circuits is well suited to sharpen frequency tuning, additional mechanisms are likely required to regulate how refined representations are amplified and expressed across cortical states.

Recent work has identified L6-CTNs as key regulators of auditory cortical state. Rather than simply modulating thalamic gain, L6-CTNs can bias cortical processing toward detection-favoring or discrimination-favoring regimes through their interactions with thalamic circuits and local inhibitory networks (Guo et al., 2017; Ibrahim et al., 2021). Reduced recruitment of L6-CTNs biases A1 toward low-gain/frequency-selective regimes in which only strongly matched sensory inputs reliably elicit population responses, favoring frequency discrimination at the expense of sensitivity. Within this framework, our finding that DE selectively weakens intracortical excitatory drive onto L6-CTNs provides a plausible, complementary mechanism through which intracortical refinement and state-dependent gain control may be coordinated in the auditory cortex. In particular, the selective reduction of excitation from L4 to L6-CTNs, reflected by a decreased E/I balance for L4-originating inputs, suggests that feedforward sensory activity conveyed through L4 is less effective at engaging L6-CTNs. Concurrent reductions in supragranular (L1–3) and infragranular (L5) excitation further limit contextual and deep-layer recruitment of L6-CTNs. These changes are consistent with a reduced probability of L6-CTN engagement during sound-evoked activity, biasing A1 toward a frequency-selective cortical state relative to normal-reared conditions. In this context, the potentiation of thalamocortical synapses onto L4 observed following DE (Petrus et al., 2014) may preserve sensitivity to sound, while reduced intracortical recruitment of L4 pyramidal cells (Meng et al., 2015, 2017a) in parallel with reduced intracortical recruitment of L6-CTNs constrains frequency selectivity.

One limitation of this study is its reliance on tdTomato labeling driven by the Ntsr1-Cre_GN220 line to identify L6 corticothalamic neurons, rather than independently confirming the projection identity of each recorded neuron using retrograde tracing or axonal reconstruction. This classification is supported by foundational anatomical studies of the same mouse line. In auditory cortex, retrograde tracer injections into the medial geniculate body labeled 97% of L6 Ntsr1-reporter-positive neurons, indicating that nearly all neurons targeted by this line possess a thalamic projection (Guo et al., 2017). Similarly, retrograde labeling from the corresponding sensory thalamic nuclei identified 82.6% of reporter-positive neurons in visual cortex and 91.0% in somatosensory cortex (Kim et al., 2014). In visual cortex, 88.2% of reporter-positive L6 neurons were labeled from the dLGN and observed no overlap between Ntsr1-Cre_GN220 reporter expression and neurons retrogradely labeled from the claustrum (Sundberg et al., 2018). Together with additional anatomical characterization demonstrating that this line preferentially targets L6 neurons whose axons innervate the thalamus rather than L6 neurons whose long-range axons remain within the cortex (Bortone et al., 2014), these findings have led the field to conventionally classify Ntsr1-Cre_GN220-labeled neurons in primary sensory cortical L6 as corticothalamic neurons. We adopt this established, genetically defined classification here. Nevertheless, prior validation at the population level does not establish the projection identity of every reporter-positive neuron recorded in the present study. Rare off-target reporter-positive cells, including a small population described near the cortical white matter, cannot be excluded completely, although our restriction of recordings to the upper half of L6 makes their inclusion unlikely (Sundberg and Granseth, 2018). Our findings should therefore be interpreted as applying to the Ntsr1-Cre_GN220-defined L6 corticothalamic population, with corticothalamic identity inferred from prior validation of the driver line rather than independently demonstrated for each neuron examined in this study.

Taken together, these findings support a model in which cross-modal deprivation produces complementary plasticity at distinct circuit nodes. Strengthening of feedforward thalamocortical pathways enhances sensory gain and lowers detection thresholds, while selective weakening of intracortical excitation onto L6-CTNs biases cortical processing toward discrimination-favoring states. Through this decoupling of feedforward gain from global cortical amplification, DE enables simultaneous enhancement of auditory sensitivity and frequency selectivity.

## Synthesis

Reviewing Editor: Luke Remage-Healey, University of Massachusetts Amherst Decisions are customarily a result of the Reviewing Editor and the peer reviewers coming together and discussing their recommendations until a consensus is reached. When revisions are invited, a fact-based synthesis statement explaining their decision and outlining what is needed to prepare a revision will be listed below. The following reviewer(s) agreed to reveal their identity: NONE. Note: If this manuscript was transferred from JNeurosci and a decision was made to accept the manuscript without peer review, a synthesis may not be available.

The authors, in their transfer rebuttal, have sufficiently addressed the major concerns of both original reviewers. In particular the focus on one level of biological analysis (whole cell recordings) and clarity of the findings do not detract from the conclusions and the study warrants publication.
